# Vertical stratification-enabled early monitoring of cotton Verticillium wilt using *in-situ* leaf spectroscopy via machine learning models

**DOI:** 10.3389/fpls.2025.1599877

**Published:** 2025-06-23

**Authors:** Yi Gao, Changping Huang, Xia Zhang, Ze Zhang, Bing Chen

**Affiliations:** ^1^ National Engineering Research Center of Satellite Remote Sensing Applications, Aerospace Information Research Institute, Chinese Academy of Sciences, Beijing, China; ^2^ University of Chinese Academy of Sciences, Beijing, China; ^3^ Xinjiang Production and Construction Corps Oasis Eco-Agriculture Key Laboratory, College of Agriculture, Shihezi University, Shihezi, Xinjiang, China; ^4^ Research Institute, Xinjiang Academy Agricultural and Reclamation Science, Shihezi, China

**Keywords:** cotton Verticillium wilt, vertical leaf layer, hyperspectral reflectance, machine learning, disease severity

## Abstract

Early monitoring of cotton Verticillium wilt (VW) is crucial for preventing significant yield losses and quality deterioration. Current hyperspectral approaches often overlook the bottom-up disease progression and the impact of leaf stratification on VW detection. To address this, vertical spectral traits were examined to improve early diagnosis. A total of 551 *in-situ* leaf spectra were averaged from thousands of measurements, alongside corresponding RGB images from top, middle, and bottom leaf layers. Five severity levels (SL=0-4) were classified based on lesion coverage. Various vegetation indices and signal features were extracted for VW identification. Three feature selection methods, Relief-F, Lasso, and Random Forest (RF), were integrated with five machine learning models, including LightGBM, ANN, XGBoost, RF, and SVM. Results showed that spectral reflectance varied significantly by severity and layer, with the most pronounced variations in the bottom layer’s visible spectrum. LightGBM with RF-selected features achieved the best performance and fastest training, with accuracies of 0.82, 0.81, and 0.91 for the top, middle, and bottom leaf layers, respectively. Early-stage detection (SL=0-2) was most effective in the lowest layer, showing 38% and 34% higher precision (SL=1) than the upper two. Critical spectral features varied with vertical leaf layers and disease severity, with blue and red-edge bands identified as most important. For assessing five disease severity levels, the most informative features for the top, middle, and bottom layers were Ant_Gitelson_, Blue Index (B), and PRI_570_. For detecting early symptoms (SL=1), the blue band was particularly effective, followed by water-related bands. At the initial infection stage, the most significant indicators for top, middle, and bottom layers were Blue/red index (BRI), B, and WSCT, respectively. This study deepens understanding of vertical leaf spectral dynamics and enables rapid, non-destructive *in vivo* detection of cotton Verticillium wilt, enhancing the applicability of portable hyperspectral devices and informing leaf-layer-aware precision disease management strategies.

## Highlights

- A dataset of 551 cotton leaf samples combining hyperspectral reflectance and high-resolution RGB images enables early Verticillium wilt (VW) detection using *in-situ* spectral analysis with vertical leaf stratification.
- LightGBM with RF-selected features achieves 91% accuracy for bottom-layer monitoring, outperforming top and middle layers by 38% and 34% in early-stage detection (SL=1).
- Key features for VW severity classification (SL=0-4) include Ant_Gitelson_, Blue Index (B), and PRI_570_. Early-stage infection (SL=1) is best detected by BRI, B, and WSCT, with the blue band (400-500 nm) important across all layers.
- 4. Three feature selection methods and five machine learning models were evaluated. LightGBM with RF-selected features showed optimal performance for early VW monitoring.

## 1 Introduction

Cotton is a significant economic crop that plays an important role in the global fiber and oilseed supply. Verticillium wilt (VW), a soil-borne fungal disease primarily caused by the pathogens *Verticillium dahlia* and *Verticillium albo-atrum*, is threatening global cotton production and quality seriously. It leads to a large economic loss. The fungal pathogens of VW naturally exist in soil and can invade the root system of cotton plants, penetrating through the cortex and xylem, ultimately causing the plants to wither and die. Traditional methods for monitoring cotton VW, such as field surveys and laboratory-based chemical analyses (e.g., PCR), are often time-consuming, spatially limited, and costly, with chemical assays typically requiring destructive sampling. In recent years, VW, an aggressive vascular disease, has shown earlier onset, faster progression, and increased severity, making timely monitoring even more challenging with conventional methods. In contrast, remote sensing enables rapid, non-destructive disease monitoring, making it a promising tool for early and accurate VW monitoring (Skendžić et al., 2023).

Owing to its high spectral resolution, hyperspectral remote sensing can detect subtle physiological changes caused by infections, offering the potential for early disease monitoring, even during the asymptomatic phase (Zhao et al., 2022b). Cotton VW infects the vascular system, turning it gray or dark brown, obstructing conductive tissues and causing leaf yellowing, wilting, bud and boll shedding, and plant death. Pathogen-infected leaves often develop spots, necrosis, or wilting, reducing pigment content and activity. This causes an increase in visible spectral reflectance (400-700nm) and a blue shift in the red-edge region (670–730 nm) (Zhang et al., 2012). Additionally, variations in plant water status due to leaf chlorosis can alter spectral reflectance patterns in the near-infrared and short-wave infrared bands. Therefore, comprehensive extraction and in-depth analysis of disease-related spectral features from leaves enable effective early monitoring of cotton VW.

Leaf-scale hyperspectral monitoring captures subtle spectral changes and provides more pure disease-related information compared to canopy-scale monitoring (Zhang et al., 2019). However, current early monitoring studies, which focus on severity levels below 50% and use 10% as a threshold (Yang et al., 2022b), still lack the capability to identify VW during the initial days of symptom onset, when only about 5% of the leaf area may be affected. Most research relies on leaf clips or controlled laboratory conditions (Bienkowski et al., 2019), which fail to represent whole-leaf conditions, introduce subjectivity in measurement point selection (Yang et al., 2024), and produce data that often differ significantly from natural field conditions (Appeltans et al., 2021). These limitations hinder the application of hyperspectral sensors for *in-situ*, rapid, and non-destructive monitoring in complex outdoor environments. Furthermore, although many studies have focused on leaf-scale disease monitoring using hyperspectral remote sensing, most rely on mixed leaf samples or top-layer leaves, neglecting the actual progression of VW in cotton, which typically starts in the bottom plant layers and moves upward (Jing et al., 2022).

In the early stages of cotton VW infected, symptoms are visible only on the bottom leaves, while the upper healthy leaves can obscure the canopy spectrum, making it challenging to capture the spectral characteristics associated with infection. This non-synchronization between the infection in lower leaves and the healthy status of upper leaves significantly affects the accuracy of VW severity estimation using canopy hyperspectral data (Li et al., 2022). This problem has become a key limiting factor to further improve the accuracy and applicability of spectral models for precise cotton VW monitoring. Numerous studies on canopy-scale hyperspectral monitoring for cotton VW have highlighted the challenge of identifying early symptoms, which primarily appear on the bottom-layer leaves (Kang et al., 2022; Ma et al., 2024; Nie et al., 2024). Therefore, considering the differences in spectral characteristics across vertical leaf layers, monitoring cotton VW with respect to leaf position offers a new perspective for early disease detection. The exploration of how VW alters hyperspectral signatures across distinct leaf layers, clarifying their variations and identifying vertical leaf layer spectral bands for disease detection, necessitates further investigation.

Current research on vertical spectral monitoring primarily focuses on the vertical distribution of plant pigments, water content, and nutrient levels (Yang et al., 2022a). Stratification strategies for such analyses are typically based on either one-third of the plant height or the average number of leaves per layer (Zhang et al., 2024). However, only a few studies have explored how vertical distribution affects disease monitoring accuracy. Approaches to disease monitoring considering vertical distribution mainly include multi-angular spectral observations and stratified leaf-layer spectral measurements. For instance, to address the challenge of lower-layer leaf occlusion in traditional vertical observations, studies have utilized multi-angular spectral parameters to determine optimal viewing angles for monitoring wheat powdery mildew, significantly improving early detection accuracy (He et al., 2021; Song et al., 2022). Additionally, Huang et al. (2015) investigated *in-situ* spectral differences among infected leaves across different layers and developed a universal spectral index for monitoring wheat stripe rust by integrating spectral features from top, middle, and bottom leaf layers, achieving high monitoring accuracy with R² of 0.88. However, current studies on spectral differences in leaves across positions are largely limited to crops such as wheat and corn, with limited research on cotton VW (Li et al., 2015; Ye et al., 2018). Only a few studies have qualitatively compared laboratory-measured spectral reflectance of VW-infected leaves, noting significant spectral variations due to leaf position (Kuligowski et al., 2006; Chen et al., 2014). Nevertheless, how these position-related spectral differences impact the monitoring of cotton VW, especially at early stages, remains unclear.

Differ from crops like wheat, cotton plants typically exhibit dense foliage, a structure that maximizes photosynthesis but also leads to significant differences in photosynthetically active radiation (PAR) received by different leaf layers (Sassenrath-Cole et al., 1996; Wang et al., 2023b). Due to shading from upper leaves, lower-layer leaves receive less light, resulting in variations in their light environment and growth conditions, which in turn affect their optical properties (Li, 2007). Ignoring the vertical heterogeneity within the canopy may compromise the accuracy of spectral reflectance characteristics, potentially leading to misinterpretations of plant physiological traits (Yang et al., 2017).

Moreover, most existing studies rely on a limited number of spectral bands to construct disease monitoring indices, which provide restricted information and fail to fully utilize the entire spectral range. Extracting comprehensive features from hyperspectral data to capture subtle spectral changes during early disease stages or under complex conditions is crucial for effective disease monitoring (Zhang et al., 2019). Features commonly used in power quality monitoring, such as peak values and kurtosis, can identify weak disturbance signals based on curve morphology, potentially aiding in the detection of early spectral changes in VW-infected cotton leaves (Bastami and Vahid, 2021). However, the fusion of multiple features often leads to redundancy, making the integration of feature selection and machine learning methods a mainstream approach in remote sensing for disease monitoring, widely applied in precision agriculture (Zhao et al., 2022a). Commonly used feature selection methods for vegetation spectral disease monitoring include Relief-F, Random Forest, and PCA (Hennessy et al., 2020), while frequently employed machine learning algorithms are ANN, SVM, and RF (Deng et al., 2019; Sanaeifar et al., 2023). As each method focuses on different aspects, their optimal combination can enhance the extraction and sensitivity to weak signal changes, having been proven effective in monitoring crop diseases (Feng et al., 2022; Zhang et al., 2023). In recent years, numerous studies have compared combinations of different disease monitoring methods (Poblete et al., 2020; Song et al., 2022). However, the choice of methods varies across different crops and disease types (Chan et al., 2020), and the optimal combination for cotton VW monitoring remains uncertain. Additionally, precision agriculture requires continuous updates to disease monitoring methods to provide higher accuracy in disease diagnosis. LightGBM, as an emerging efficient machine learning algorithm, offers advantages in time, memory usage, accuracy, and interpretability, making it particularly suitable for timely, precise, and efficient disease monitoring (Harumy et al., 2024). It has been widely applied in medical disease prediction with outstanding performance (Wang et al., 2023a), but its application in plant disease monitoring remains limited. The potential of LightGBM for cotton VW monitoring, its advantages over traditional models, and its applicability in field environments require further exploration.

This study aims to investigate how vertical leaf stratification affects the hyperspectral response of cotton Verticillium wilt, with a particular focus on *in-situ* early monitoring. Based on the above analysis, this study focuses on the monitoring effectiveness of VW across vertically stratified cotton canopies. With non-destructive hyperspectral data from whole leaves, an optimal combination of feature selection approaches and machine learning models is employed to achieve early VW detection and evaluate the impact of leaf position on detection performance. This research addresses the gap in understanding how different leaf positions influence cotton VW monitoring. To the best of our knowledge, this study pioneers the characterization of early spectral signatures in VW-infected cotton stem leaves under field settings, aiming to meet the demand for rapid *in-situ* monitoring in natural environments. The main contributions of this study are as follows: (1) constructing a comprehensive dataset of 551 samples, including high-resolution RGB images and averaged spectra from 2,900 individual spectral measurements of cotton main-stem leaves, to enable the stratified early monitoring of cotton VW; (2) comparing the performance of various feature selection methods (Relief-F, Lasso, and RF) and machine learning models (LightGBM, ANN, XGBoost, RF, and SVM) in monitoring cotton VW; (3) assessing the influence of leaf position within cotton plants on early VW detection performance.

## 2 Materials and methods

### 2.1 Overall framework of the study

**Figure 1:**
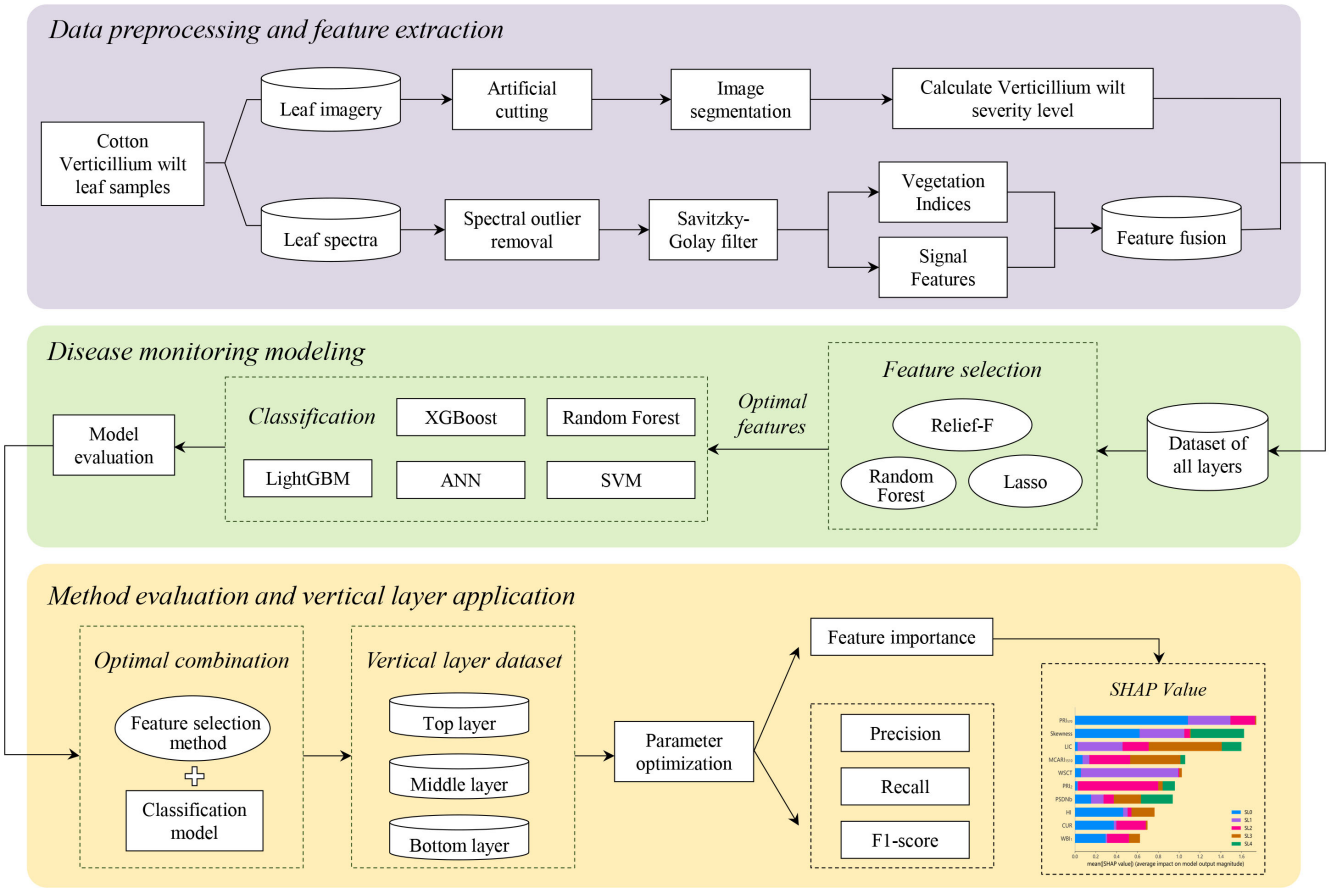
Hyperspectral-based vertical leaf layer monitoring framework for cotton Verticillium wilt.

This study establishes a framework for monitoring VW across vertically stratified cotton leaf layers based on hyperspectral data. **Figure 1** illustrates the workflow encompassing *in-situ* hyperspectral data acquisition, vertical leaf layer feature optimization, and machine learning model construction, delivering layer-specific diagnostic precision across top, middle, and bottom leaf layers.

### 2.2 Field experiments

**Figure 2:**
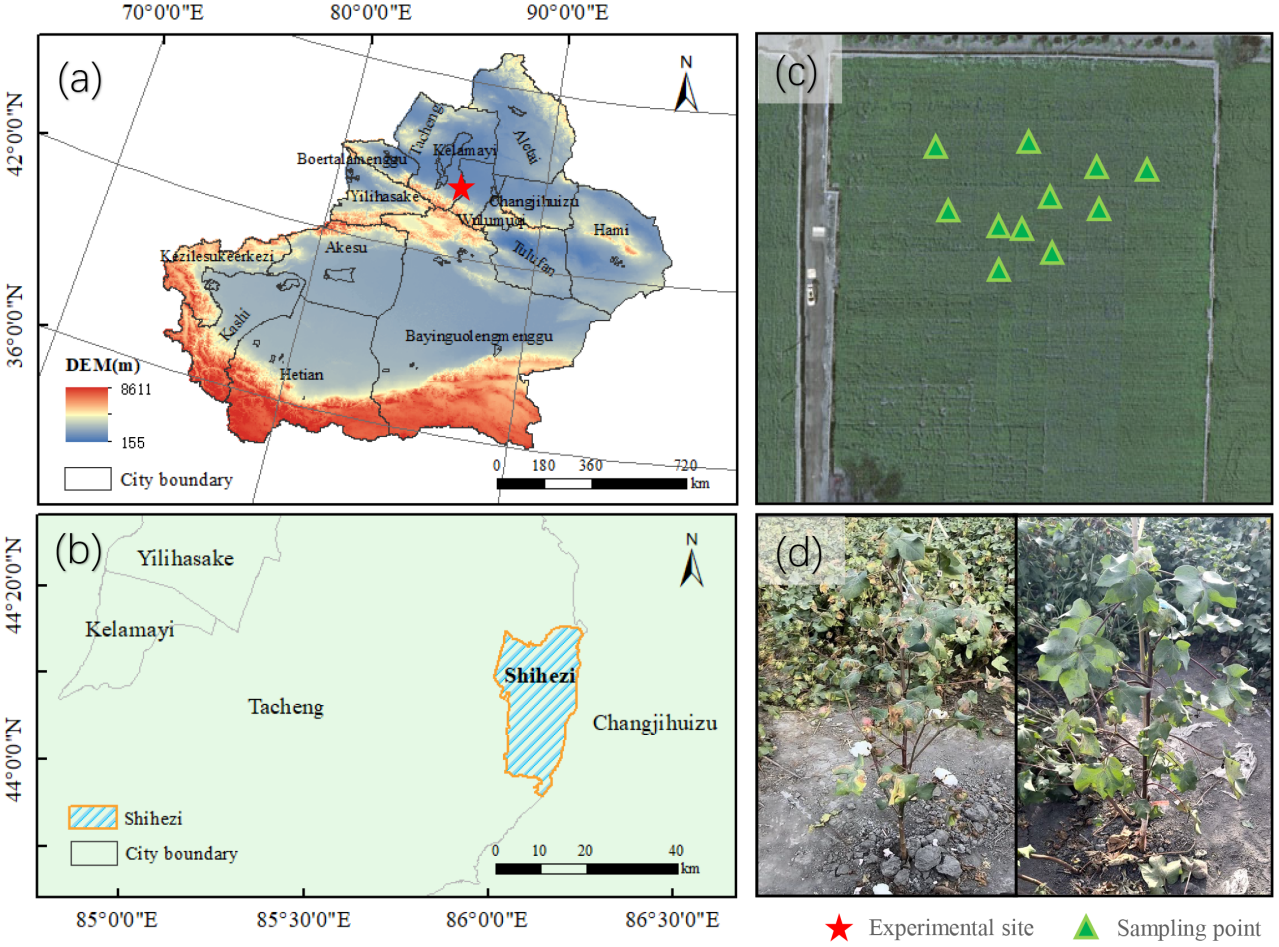
Experimental area at the Cotton Research Institute, Shihezi Academy of Agricultural Sciences, Shihezi City, Xinjiang. **(a)** Xinjiang Uygur Autonomous Region (red star: Shihezi City); **(b)** Administrative boundary of Shihezi City (blue shaded area); **(c)** Field sampling layout within VW-infected plots; **(d)** Symptomatic cotton plants with VW infection.

The trial was conducted in the field of VW disease at the Shihezi Institute of Agricultural Sciences (44.33°N, 86.05°E), Xinjiang (**Figure 2**). As Xinjiang is the most important cotton planting region in China, accounting for 23.1% of global cotton output and 90.2% of China’s cotton production (Feng et al., 2024). The region has a typical temperate continental climate with abundant sunlight. The national-level Verticillium wilt experimental field is cultivated with multiple candidate cotton germplasm lines and is artificially inoculated with the VW pathogen each year to maintain consistent disease pressure. Sowing was conducted from April 15-20, 2023, using ridge film mulching with on-film hole sowing and subsurface drip irrigation. The planting configuration followed a row spacing pattern of 10 + 66 + 10 + 66 cm. Irrigation was applied at intervals of 8–10 days, totaling 10–12 applications throughout the crop growth period. Fertilization included mono-ammonium phosphate (MAP) and urea, with potassium primarily supplied as available potassium (KCl), and potassium dihydrogen phosphate (KH₂PO₄) applied as a topdressing during the late growth stages. All fertilizers were dissolved in water and delivered via drip irrigation. The field is protected from external interference, and is irrigated and fertilized regularly to prevent drought stress or other stress that could affect VW monitoring.

The experiments were conducted from August 4th to September 10th, 2023. This period is the peak of cotton VW and covers the key growth period of flowering stage, experiencing the significant process from reproductive stages to vegetative stages. In this study, we randomly selected 11 distinct cotton plants from the experimental field and collected main stem leaves for analysis. Between August 4th and 10th, eleven cotton plants that had not shown symptom of VW were selected in the field. The typical main stem leaves at different leaf positions of these plants were used as experimental subjects to observe the full progression of the disease, from healthy to symptomatic, followed by leaf yellowing and senescence. 

### 2.3 Data collection

#### 2.3.1 Leaf spectral reflectance and image acquisition

This study primarily collects hyperspectral reflectance and corresponding high-resolution RGB images of cotton main-stem leaves. *In-situ* spectral measurements of the main stem leaves were conducted using a Spectral Evolution R PSR+ 3500 hand-held spectroradiometer (Spectral Evolution Inc., Lawrence, MA, USA). The spectroradiometer measures spectral wavelengths ranging from 350 to 2500 nm, with spectral resolutions of 3.5 nm at 350–1000 nm, 10 nm at 1500 nm, and 7 nm at 2100 nm, at 1nm intervals. To ensure the entire leaf remained within the probe’s field of view, the leaf, while still attached to the cotton plant, was gently flattened against a horizontally placed diffuse matte blackboard, which served as the measurement background, and measured at an orthophoto angle. The distance between the probe and the leaf was approximately 9–15 cm, adjusted flexibly based on pre-calculated distances corresponding to the leaf size, giving the conical field of view of 25°. A white reference panel, placed in the same scene as the leaf, was used for spectroradiometer calibration every 10–15 minutes to account for variation in incident light intensity. Measurements were taken on every sunny day with minimal or no cloud cover, at times when sunlight was strong and stable.

An RGB image was captured immediately after each spectral measurement at an orthophoto angle using an iPhone 14 Pro (Apple Inc., Cupertino, CA, USA), equipped with a 48 MP main camera featuring a quad-pixel sensor and producing images with a resolution of 8064×6048 pixels. The proportion of VW-infected areas and the color changes of the leaf in the images were used to determinate the disease severity.

A total of 2,900 *in-situ* hyperspectral measurements were collected from 33 cotton main-stem leaves under field conditions, alongside 551 nearly synchronous RGB images. Following spectral preprocessing and averaging, 551 whole-leaf spectra were derived from different leaf positions, which covers the different severities of cotton VW.

#### 2.3.2 Division of cotton VW severity levels

**Figure 3:**
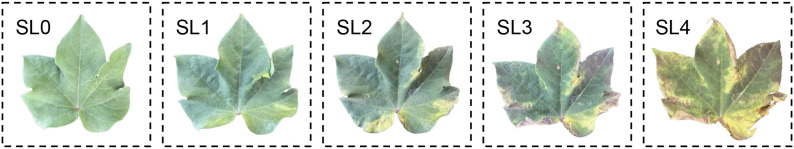
Cotton leaves with different VW severity level.

The diseased-to-total pixel ratio within Regions of Interest (ROI) was calculated to quantify infection severity, using the delineation of diseased leaf areas as ROIs. Based on long-term field observations of symptom progression, it was observed that healthy and early-stage diseased states persist longer than the mid-to-late stages, where the disease progresses slowly in the early stages and rapidly in the later stages. Furthermore, when lesions exceed 50% coverage, extensive pathogen invasion have led to irreversible damage (Yang et al., 2022b). Considering these factors and in accordance with previous studies, where cotton VW severity was assessed by increasing the disease level with each 1/4 increase in leaf incidence (Chen et al., 2011), disease severity was classified into five progressive levels (SL=0-4). These levels were defined using lesion coverage thresholds of 0%, 5%, 25%, 50%, and 100%, corresponding to the healthy, slightly infected, mildly infected, moderately infected, and severely infected stages. In the slightly infected stages of disease, cotton leaves exhibit mild symptoms with localized yellowing between the veins. As the disease progresses, significant damage occurs to the localized palisade and spongy mesophyll cells, leading to tissue deformation. The infection spots then gradually expand, accompanied by leaf margin scorching and curling. In severe cases, the infected spots spread to nearly half of the leaf, followed by distortion of the leaf shape. In extreme cases, the entire leaf turns yellow, curls, and ultimately dies. The changes in disease severity of a leaf sample in a cotton plant, from healthy to wilted and dead, are shown in **Figure 3**.

Based on the division of cotton VW severity mentioned above, 173 health samples (SL0) were identified, along with 378 symptomatic samples classified as SL=1-4. To the best of our knowledge, this is the first time that the *in-situ* leaf changes at the initial symptom appearance stage (SL1) of VW have been captured.

#### 2.3.3 Vertical stratification of cotton leaf layers

Due to individual differences among cotton plants, the number of leaves on the main stem is not consistent, meaning leaf position numbers are not identical. Therefore, each of the eleven cotton plants was divided into three layers based on the bottom, middle, and top thirds of the total number of typical main stem leaves from bottom to top (Zhang et al., 2024). The whole dataset was accordingly divided into three subsets: top layer, middle layer, and bottom layer, to analyze the differences in spectral features across leaf positions for monitoring cotton VW. The sample distribution across the three subsets includes 215 samples in the top layer, 214 in the middle layer, and 122 in the bottom layer, as shown in **Table 1**.

**Table 1:** Division of cotton leaf VW severity level.

SL level	Severity	Disease spot	Sample size			
			Top layer	Middle layer	Bottom layer	All layers
SL0	Healthy	0%	63	60	50	173
SL1	Slightly infected	0-5%	29	41	23	93
SL2	Mildly infected	5-25%	51	52	26	129
SL3	Moderately infected	25-50%	53	48	16	117
SL4	Severely infected	50-100%	19	13	7	39

### 2.4 Hyperspectral data analysis and feature extraction

#### 2.4.1 Spectrum preprocessing

**Figure 4:**
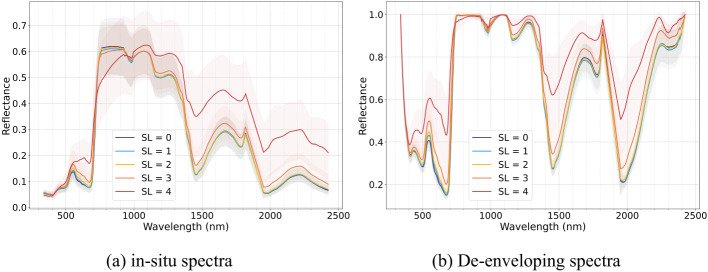
Leaf raw spectral curve **(a)** and de-enveloping spectral curve **(b)** of Verticillium wilt (VW) at different severity levels across all leaf layers.

To minimize noise at the ends of the spectral reflectance curve and avoid signal loss due to light absorption by atmospheric water vapor, the spectral range was restricted from 340–2500 nm to 340–1820 nm and 1950–2420 nm. Individual outlier spectra, identified from the primary dataset of 3,617 raw spectral measurements with repetitions, were removed due to strong interference from external factors. A total of 2,900 high-quality spectra were retained for subsequent analysis in this study. Then, the remaining spectra were processed using a Savitzky-Golay smoothing filter to mitigate noise interference. Mean leaf spectral reflectance for each disease severity level (SL=0-4) was calculated to evaluate their spectral response (**Figure 4**).

#### 2.4.2 Vegetation indices construction

The nutritional, physiological, structural, and water content traits of crops change after pathogen infection, leading to alterations in reflectance. A total of 34 vegetation indices (VIs), commonly used in studies on vegetation disease monitoring, were constructed based on leaf reflectance. These indices encompass 11 categories, including those related to pigment content (e.g., chlorophyll a+b, carotenoids and xanthophylls), photosynthetic activity (e.g., fluorescence indices), leaf structure, disease, water content and blue-green-red ratios (**Table A1**).

**Table A1:** Hyperspectral vegetation indices utilized in this study.

Vegetation indices	Equation	Reference
Structural indices		
Red-edge NDVI	$RNDVI=(R_{750}-R_{705})/(R_{750}+R_{705})$	Barnes et al. (2000)
Green NDVI	$GNDVI=(R_{750}-R_{540}+R_{570})/(R_{750}+R_{540}-R_{570})$	Gitelson and Merzlyak (1997)
Xanthophyll indices		
Photochemical Reflectance Index (570)	$PRI_{570}=(R_{570}-R_{531})/(R_{570}+R_{531})$	Gamon et al. (1992)
Photochemical Reflectance Index (600)	$PRI_{600}=(R_{600}-R_{531})/(R_{600}+R_{531})$	Gamon et al. (1992)
Photochemical Reflectance Index (515)	$PRI_{515}=(R_{515}-R_{531})/(R_{515}+R_{531})$	Hernández-Clemente et al. (2011)
Anthocyanin & Carotenoid		
Anthocyanin (Gitelson)	$A{\text{nt}}_{Gitelson}=(1/R_{550}-1/R_{700})\times R_{780}$	Gitelson et al. (2003)
Carotenoid Reflectance Index (550_515)	$CRI_{550\_515}=(1/R_{515})-(1/R_{550})$	Gitelson et al. (2006)
Carotenoid Reflectance Index (700_515)	$CRI_{700\_515}=(1/R_{515})-(1/R_{700})$	Gitelson et al. (2006)
Chlorophyll a+b		
Chlorophyll Index Red Edge	$CI=R_{750}/R_{710}$	Haboudane et al. (2002)
Vogelmann Index	$VOG_{1}=R_{740}/R_{720}$	Vogelmann et al. (1993)
	$VOG_{2}=(R_{734}-R_{747})/(R_{715}+R_{720})$	Vogelmann et al. (1993)
Gitelson and Merzlyak Index	$GM=R_{750}/R_{700}$	Gitelson and Merzlyak (1996)
Chlorophyll b	$PSDN_{b}=(R_{800}-R_{635})/(R_{800}+R_{635})$	Blackburn (1998)
Transformed Chlorophyll Absorption in Reflectance Index	$TCARI=3\times [(R_{700}-R_{670})-0.2\times (R_{700}-R_{550})\times (R_{700}/R_{670})]$	Haboudane et al. (2002)
Modified Chlorophyll Absorption Reflectance Index	$MCARI=[(R_{701}-R_{671})-0.2\times (R_{701}-R_{549})]/(R_{701}/R_{670})$	Daughtry (2000)
Reflectance Band Ratio Index	$DCabC\text{xc}=R_{672}/(R_{550}\times (3\times R_{708}))$	Datt (1998)
R/G/B indices		
Blue Index	$B=R_{450}/R_{490}$	Calderón et al. (2013)
Blue/red Index	$BRI=R_{450}/R_{690}$	Zarco-Tejada et al. (2012)
Lichtenthaler Index	$LIC=R_{440}/R_{690}$	Lichtenthaler (1996)
Blue Fraction	$BF=R_{400}/R_{410}$	Zarco-Tejada et al. (2018)
Fluorescence		
Fluorescence Ratio Index	$FRI=R_{690}/R_{630}$	Zarco-Tejada et al. (2000)
Fluorescence Curvature Index	$FCI=R_{683}^{2}/(R_{675}\times R_{691})$	Zarco-Tejada et al. (2000)
Reflectance Curvature Index	$CUR=(R_{675}\times R_{690})/R_{683}^{2}$	Zarco-Tejada et al. (2000)
Norm. Diff. N. Index	$NDNI=\log(1/R_{1510})-\log(1/R_{1680})/(\log(1/R_{1510})+\log(1/R_{1680}))$	Serrano et al. (2002)
Photochemical Reflectance Index	$PRI_{1}=R_{685}/R_{655}$	Meroni et al. (2009)
	$PRI_{2}=R_{680}/R_{630}$	Meroni et al. (2009)
Water content		
Water Band Index	$WBI_{1}=R_{970}/R_{900}$	Penuelas et al. (1997)
	$WBI_{2}=R_{1150}/R_{1450}$	Sapes et al. (2022)
Water Stress and Canopy Temperature	$WSCT=(R_{970}-R_{850})/(R_{970}+R_{850})$	Babar et al. (2006)
Normalized Difference Water Index	$NDWI_{1}=(R_{835}-R_{1610})/(R_{835}+R_{1610})$	Gao (1996)
	$NDWI_{2}=(R_{860}-R_{1195})/(R_{860}+R_{1195})$	Gao (1996)
Plant disease index		
Healthy-index	$HI=(R_{534}-R_{698})/(R_{534}+R_{698})-R_{704}/2$	Mahlein et al. (2013)
Nitrogen & Other pigment indices		
Carter Index	$CTRI_{1}=R_{695}/R_{420}$	Carter (1994)
Modified Chlorophyll Absorption Reflectance Index (1510)	$MCARI_{1510}=[(R_{700}-R_{1510})-0.2\times (R_{700}-R_{550})]/(R_{700}/R_{1510})$	Herrmann et al. (2010)

#### 2.4.3 Signal features calculation

Signal features evaluate electromagnetic spectrum variations from statistical and morphological perspectives. The differences in distance and morphological information between spectral curves are useful for measuring the heterogeneity between healthy and diseased plants. Some studies have successfully applied information entropy for band selection to differentiate between healthy and diseased plants based on spectral data (Deng et al., 2019). Drawing from the literature on power quality monitoring and abnormal signal detection in bearing faults, this study extracted 13 signal features from the spectral curves of cotton leaves based on mathematical methods and optimization techniques. These features include Kurtosis, Entropy, Fractal Dimension, Peak Factor, Pulse Factor, Crest Factor, Energy Ratio, Spectral Flatness, Mean, Variance, Skewness, Peak Vibration, and RMS Vibration (Shannon, 1948; Cooley and Tukey, 1965). These features characterize the statistical distribution, energy dynamics, and structural complexity of the spectral curves, providing comprehensive insights into the signal patterns associated with cotton leaf reflectance infected by VW.

### 2.5 Feature selection and classification models

#### 2.5.1 Feature selection methods

VW infection in cotton impairs photosynthesis, induces chlorosis, and disrupts water transport, leading to the changes in spectral reflectance. VIs derived from hyperspectral data can effectively capture these physiological alterations. On the other hand, signal features (SFs), which reflect morphological changes in the spectral curve, offer valuable insights into the disease’s progression. By using VIs and SFs as input features, the disease can be detected from both local and global perspectives, providing a more comprehensive method for monitoring the onset and development of cotton VW.

To prevent the high redundancy in the original data and improve feature selection efficiency, three feature selection methods (Relief-F, Lasso and RF) commonly used in remote sensing disease monitoring literature were employed to obtain feature importance for VW monitoring (Sanaeifar et al., 2023). Relief-F ranks features based on their ability to distinguish between similar instances, making it useful for high-dimensional data, though it may struggle with highly correlated features (Zhang et al., 2019; Wu et al., 2023). Lasso, a linear regression method, performs both variable selection and regularization by shrinking less important feature coefficients to zero, thus preventing overfitting (Yang et al., 2024). Random Forest, an ensemble learning method, assesses feature importance by evaluating how each feature contributes to model performance, effectively handling non-linear relationships and interactions. Its inherent robustness enables it to manage complex, noisy, high-dimensional datasets effectively (Huang et al., 2022). To ensure that all input features were given equal consideration, the data were normalized before being used in the models.

#### 2.5.2 Machine learning-based classification models

To evaluate model performance in monitoring VW disease, five machine-learning algorithms were employed, including LightGBM, XGBoost, Random Forest, Support Vector Machine (SVM) and Artificial Neural Network (ANN) (Zheng et al., 2023). LightGBM uses the gradient-based one side sampling (GOSS) and exclusive feature bundling (EFB) algorithm to optimize the handling of category features. By combing sparse features and bundling mutually exclusive features, it further optimizes the training speed compared to other gradient boosting decision tree (GBDT) models. Considering computational efficiency, memory usage, and predictive performance, LightGBM is a highly suitable algorithm for hyperspectral analysis due to its fast training speed and strong interpretability, making it well-suited for capturing early spectral signals and supporting timely monitoring of VW disease. XGBoost, though more computationally intensive, offers fine-grained control through adjustable hyperparameters, enabling the modeling of complex feature interactions. Random Forest is known for its robustness to noise and capacity to manage high-dimensional feature sets, which is advantageous for disease monitoring across heterogeneous spectral datasets. Support Vector Machine (SVM) is particularly effective in handling high-dimensional data spaces and is often recommended when dealing with limited sample sizes. Artificial Neural Networks (ANNs) are capable of capturing nonlinear relationships in spectral data, and are theoretically suited to model subtle patterns associated with early-stage disease development.

For each model, the dataset was randomly split into 70% for training and 30% for testing, and the hyperparameters were optimized using Bayesian optimization. A total of 15 combinations of feature selection methods and classifiers were evaluated based on test set precision and runtime efficiency. Based on a comprehensive performance comparison, the optimal combination of feature selection method and classifier was determined using the full dataset and subsequently applied to the three stratified subsets to evaluate its effectiveness across different vertical leaf layers.

#### 2.5.3 Performance evaluation of different models

To assess the effectiveness and efficiency of the models, evaluation metrics including precision, recall, and F1-score were calculated for each classification model. Moreover, confusion matrices were used to evaluate the classification performance of different models, with particular focus on misclassification and omission errors across disease severity levels. By analyzing the distribution of true positives, false positives, false negatives, and true negatives, the confusion matrix allows for a detailed assessment of how each model performs for individual classes.

Additionally, the Shapley Additive Explanations (SHAP) method was utilized to assess each feature significance and quantify their contributions in monitoring cotton WV across different leaf layers. All comparative experiments in this study were conducted using the Python programming language, with the Scikit-learn machine learning library employed for model development and training.

## 3 Results

### 3.1 Spectral characteristics of infected cotton leaves

The occurrence of VW leads to a reduction in leaf pigment content, destruction of leaf cell structures, and a decrease in leaf water concentrations. These physiological changes are reflected in the spectral reflectance through light absorption and scattering interactions (Huang et al., 2018). In view of all layers, as shown in **Figure 4**, the reflectance of cotton leaves consistently increased with disease severity from SL0 to SL4. As the disease progressed, the spectral differences between adjacent disease stages gradually expanded. Additionally, the envelope-removed spectra revealed that the absorption valleys across all spectral regions became more gradual with increasing disease severity, underscoring the progressive spectral alterations across all leaf layers. Compared to raw spectra, the envelope-removed spectra more effectively accentuated absorption-reflection features by eliminating background absorption effects, resulting in more distinct spectral patterns. The visible (400–700 nm) and red-edge region (≃ 705 nm) primarily reflected the degradation of the chlorophyll as VW infection progresses. In the blue (450–485 nm) and green regions (495–570 nm), the degradation of carotenoids and the accumulation of anthocyanins, respectively, become more evident with the severity of VW increased (Camino et al., 2021). VW-induced leaf structural degradation alters leaf thickness and dry matter content, impacting spectral reflectance in the red-edge and near-infrared plateau regions. Additionally, the absorption valleys in the near-infrared and short-wave infrared regions became progressively shallower, influenced by the decreasing leaf water concentration as the disease increased, leading to increased spectral reflectance.

**Figure 5:**
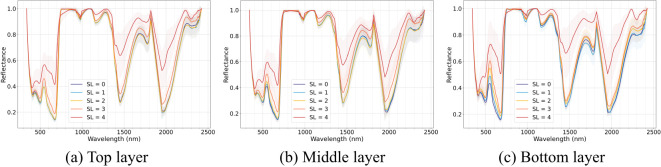
Leaf de-enveloping spectral curve of Verticillium wilt (VW) at different severity levels across different leaf layers: **(a)** top layer, **(b)** middle layer, and **(c)** bottom layer.

**Figure 5** illustrates the envelope-removed spectra of cotton leaves from different vertical layers (top, middle, and bottom) under different disease severities. Within the same disease severity level, spectral reflectance exhibits a decreasing trend as leaf layer moves higher. This trend is less pronounced in the near-infrared (NIR) region but becomes more apparent in the visible spectral region. Spectral reflectance increased with disease severity across all layers, with differences between adjacent severity stages becoming more distinct. Absorption positions were minimally influenced by leaf position, and the results align with those observed in the non-stratified dataset. However, notable differences in spectral patterns among layers were observed. From the top to the bottom layers, differences between disease severities became more pronounced under the same disease classification criteria. In the top layer, spectral differences between healthy, slightly and mildly infected disease stages were less evident, while differences between these stages and later stages (moderate and severe) were more distinct. As leaf position moved downward, spectral differences between healthy and mild disease stages gradually increased, along with those between moderate and severe stages. Among the three layers, the bottom layer exhibited the most prominent spectral differences across disease severities, followed by the middle layer, with the top layer showing the smallest differences, particularly under lower severity levels (SL=0-2).

### 3.2 Classification of disease severity using multi-layered dataset

**Figure 6:**
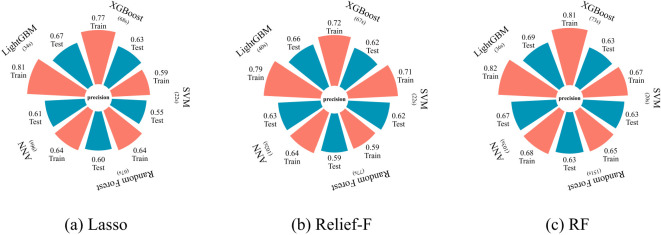
Model performance for different machine learning models based on different feature selection methods: **(a)** Lasso, **(b)** Relief-F, and **(c)** RF.

As shown in **Figure 6**, combinations of feature selection methods and classifiers exhibit significant variations in accuracy and computational efficiency for cotton VW monitoring. The RF-LightGBM combination achieves optimal performance, with a test accuracy of 0.69, surpassing other models. Specifically, RF-based feature selection outperforms Lasso and Relief-F, although Lasso and Relief-F demonstrated strong performance in specific models. When integrated with RF, LightGBM attains the highest training accuracy (0.82) and test accuracy (0.69), demonstrating its balance of precision and generalization. LightGBM also maintains stable test accuracies (0.66 and 0.67) when paired with Relief-F and Lasso, respectively, confirming its adaptability. While ANN under RF selection achieves comparable test accuracy (0.67), its runtime (103 seconds) triples that of LightGBM (36 seconds) and exists potential overfitting risks in small-sample scenarios. By contrast, XGBoost, SVM, and RF models exhibit lower test accuracies (<0.65) and prolonged training times. These results underscore the critical role of method compatibility in optimizing VW monitoring. The RF-LightGBM framework, with its high accuracy and efficiency, proves to be the most effective solution for precise, real-time monitoring of cotton leaf VW in field conditions. 

### 3.3 Stratified disease identification of cotton VW

Models for cotton VW monitoring were constructed across the top, middle, and bottom leaf layers using the RF feature selection method combined with the LightGBM classifier, as listed in **Table 2**. The results revealed notable differences in model performance between the different leaf layers, with the bottom layer achieving the highest monitoring accuracy. This indicates that disease monitoring accuracy is influenced by the vertical stratification of cotton leaves.

**Table 2:** Analysis result for cotton VW monitoring from different leaf positions.

SL level	Top layer			Middle layer			Bottom layer		
	Precision	Recall	F1-score	Precision	Recall	F1-score	Precision	Recall	F1-score
SL0	1.00	0.95	0.97	0.94	0.94	0.94	0.94	1.00	0.97
SL1	0.60	0.67	0.63	0.62	0.83	0.71	0.83	0.71	0.77
SL2	0.63	0.80	0.71	0.71	0.62	0.67	0.78	0.88	0.82
SL3	0.86	0.75	0.80	0.77	0.71	0.74	1.00	0.75	0.86
SL4	1.00	0.67	0.80	1.00	0.75	0.86	1.00	1.00	1.00
Model accuracy	0.82	0.77	0.78	0.81	0.77	0.78	0.91	0.87	0.88

**Figure 7:**
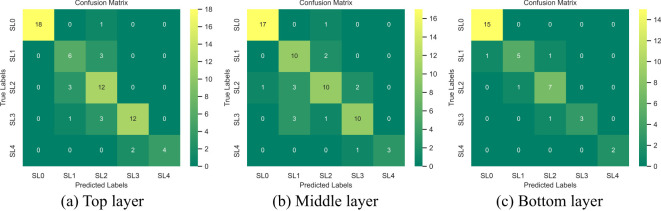
Confusion matrix of top **(a)**, middle **(b)**, and bottom **(c)** leaf positions at different disease severities (SL=0-4).

The accuracy (precision) of the top layer model reached 0.83, while the middle layer performed similarly with an accuracy of 0.81. In contrast, the bottom layer achieved the highest accuracy of 0.91, making it the most effective for monitoring VW. The confusion matrices for different disease stages across the top, middle, and bottom leaf layers are shown in **Figure 7**. For the monitoring of healthy, moderately infected, and severely infected leaves (SL=0, 3-4), the models across all three layers demonstrated high accuracy, indicating their effectiveness in identifying both healthy and advanced disease symptoms. For SL0 (healthy), the accuracy in the top, middle, and bottom layers were 1.00, 0.94, and 0.94, respectively. For SL4 (severely infected), all three layers achieved an accuracy of 1.00. However, when it comes to disease early-stage monitoring (SL1, slightly infected; and SL2, mildly infected), the performance varied significantly across layers. The bottom layer showed better accuracy for early-stage monitoring compared to the top and middle layers. The precision for SL1 (slightly infected) and SL2 (mildly infected) in the bottom layer reached 0.83 and 0.78, respectively.

The bottom leaf layer had the highest accuracy for monitoring cotton leaf VW across all disease severities. It’s performance in early disease stages also significantly outperformed the top and middle layers, highlighting the critical role of bottom leaves in early-stage disease monitoring. Additionally, the data used for modeling were derived from diverse cotton genotypes, further demonstrating the model’s robustness.

### 3.4 Layer-specific feature significance in cotton VW monitoring

**Figure 8:**
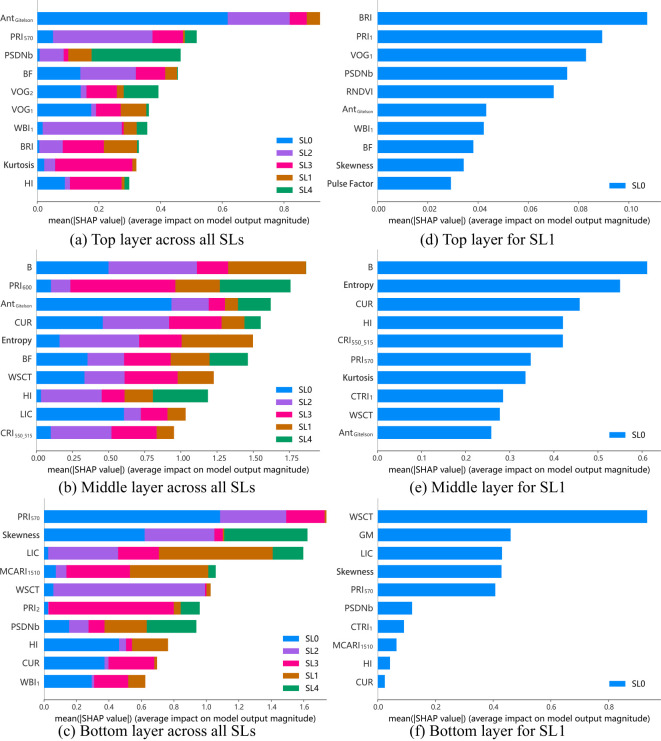
SHAP plots for the top, middle, and bottom layers at different disease severity levels (SL=0-4) **(a-c)** and at the Slightly Infected Stage (SL1) **(d-f)**.

The importance of key features for monitoring cotton VW severity across different leaf layers and stages of disease development as shown in **Figure 8**. For different disease severities, the top layer is primarily dominated by anthocyanin (Ant_Gitelson_). This is followed by PRI_570_, PSDNb and BF, which are closely linked to xanthophyll cycle, chlorophyll content and the blue band. In the middle layer, the blue band index (B), xanthophyll index (PRI_600_), and anthocyanin index (Ant_Gitelson_) are the most critical features. For the bottom layer, also xanthophyll index (PRI_570_) plays a leading role, followed by the Skewness and the blue/red index (LIC). These findings indicate that anthocyanin, xanthophyll, and the blue band are effective features for VW monitoring across the top, middle, and bottom leaf layers. However, there are also distinct differences in features across leaf layers, due to variations in nutrient and light distribution across leaf layers, as well as differences in the physiological activities of leaves at distinct positions.

In the context of slightly infected monitoring, the importance of specific features across the three layers is also shown in **Figure 8**. For the upper leaves, features representing the blue band, fluorescence, and chlorophyll content are key, with indices such as BRI, PRI_1_, VOG_1_, and PSDNb being the most critical. In the middle layer, blue band index (B) dominate, supported by entropy, and fluorescence index (CUR). For the bottom leaves, features related to water content (e.g., WSCT), Gitelson & Merzlyak (GM) and blue/red index (LIC) are critical. These findings highlight that the blue band is important in all three layers, and that key features for early monitoring across all layers are primarily related to pigments. Additionally, features linked to water content play an important role in VW early monitoring in the bottom layer.

In addition, signal features play a significant role in VW monitoring in middle and bottom layer, with entropy, and skewness ranking second, and fourth in importance for the middle, and bottom layers, respectively, as indicated by SHAP values. These signal features provide new perspectives for monitoring cotton VW, particularly during the slightly infected stage, by offering additional insights beyond traditional spectral indices.

## 4 Discussion

### 4.1 Effectiveness of stratified monitoring for cotton VW

The common approach for disease monitoring in cotton using remote sensing involves conducting spectral measurements on top layer leaves or mixed leaf samples. However, by the time disease is detected in top-layer leaves, it has often already reached an advanced stage, and the impact of leaf position on monitoring accuracy is overlooked. Considering the bottom-up spatial progression of VW, capturing spectral information from bottom leaves provides a more accurate representation of the practical early stage of infection in the cotton plant. This critical aspect is often ignored in existing studies (Lu et al., 2023). Affected by precipitation and temperature, the peak incidence period of VW in cotton in Xinjiang occurs between July and September each year. During this period, there are significant differences in pigment content among leaves at different positions, especially when nutrient transport is obstructed due to VW infection (Luo, 2010), which further exacerbates the differences between leaf positions. Additionally, the photosynthetic rate and nutrient allocation differ among leaves at different positions. These factors lead to spectral differences between leaves at different positions, which in turn affect the performance of cotton VW monitoring based on leaf spectra. Therefore, this study conducts experiments during the period when VW is prone to outbreaks, based on *in situ* leaf spectra from field experiments, focusing on analyzing the impact of different leaf layers on the monitoring of cotton VW.

As the disease progresses, lower leaves exhibit a more pronounced increase in spectral reflectance particularly in the visible region, and achieve higher VW monitoring accuracy compared to upper layers. These differences may be attributed to variations in light conditions and leaf age. On one hand, the distinct spectral responses among leaf layers originate from differences in light environments. The dense foliage architecture of cotton canopies, coupled with phototropism-driven leaf orientation, facilitates preferential PAR (photosynthetically active radiation) interception by middle and upper leaves, while creating persistent shading effects on lower canopy layers (Bertheloot et al., 2008). Longer wavelength light, with its stronger diffraction ability, is more likely to penetrate deeper into the canopy, while shorter visible wavelengths are predominantly intercepted by upper leaves (Li et al., 2022). Consequently, lower leaves adapted to low-light conditions develop thinner palisade tissues and lower chlorophyll a to b ratios (Yang et al., 2022a). This renders their spectral reflectance in the visible region potentially more sensitive to disease-induced chlorophyll degradation. On the other hand, age-related physiological decline exacerbates the vulnerability of lower leaves. Older lower leaves exhibit reduced photosynthetic efficiency (P_max_), accelerated Rubisco degradation, and weakened stress resilience compared with younger upper leaves (Sassenrath-Cole et al., 1996). When VW infection occurs, pathogen effectors such as PevD1 further disrupt lower-leaf physiology by targeting senescence-associated genes (e.g., *GhORE1*), accelerating chlorophyll breakdown and cellular disorganization (Zhang et al., 2021), which amplify spectral variations under VW infection. In contrast, upper leaves with thicker cuticles, higher chlorophyll reserves, and active metabolic repair mechanisms tend to exhibit a “threshold effect” when facing damage from the VW pathogen, requiring larger lesion areas to trigger significant spectral changes. This physiological buffering may reduce spectral discriminability in upper layers during early infection stages. Thus, the synergistic effects of light limitation, age-related physiological decline, and pathogen-driven senescence in lower leaves may enhance spectral reflectance changes, enabling more precise VW severity monitoring in lower-canopy layers compared to upper ones.

Comparing the early (SL=0-2, including healthy, initial symptomatic, and early stages) and late (SL=3-4, including mid- and late stages) monitoring performance across top, middle, and bottom layers, late-stage monitoring showed consistently high accuracy across all layers. In contrast, monitoring during the slightly infected stage showed that the monitoring performance for VW in the bottom layer was significantly better than in the top and middle layers. Comparing spectral differences across leaves with varying disease severity shows that the spectral variations in the bottom leaves are greater than those in the middle and top leaves, which is particularly evident in the visible spectral region. The spectral reflectance in the visible bands is mainly influenced by pigments, especially chlorophyll content. The chlorophyll content varies significantly across leaf layers (Yang et al., 2022a). This vertical heterogeneity in pigment distribution leads to differences in key features for VW monitoring and affects the performance of monitoring models. Under VW infection, nutrient supply is remobilized from older bottom leaves to younger top leaves (Parkash et al., 2023). Consequently, even when leaves are classified into the same disease severity level based on lesion coverage in this study, the overall health condition of the bottom leaves is noticeably poorer than that of the top and middle leaves. Cotton bottom leaves tend to appear more yellow, with lower chlorophyll content, and have reduced photosynthetic activity (Parkash et al., 2023). Therefore, the spectral differences across bottom leaves with varying disease severity, particularly in the visible region, are more evident compared to those observed in middle and top leaves. At the mid-to-late stages of infection, extensive fungal invasion results in substantial physiological and optical changes in leaves across all layers. These changes become more uniform at severe disease stages, facilitating consistent VW monitoring regardless of leaf position. These findings demonstrate that bottom layer leaves have a distinct advantage in the monitoring of VW, especially during the slightly infected stages of the disease, compared to upper and middle leaves.

### 4.2 Critical spectral features for assessing disease severity

The key features for monitoring VW in cotton, especially vegetation indices derived from the spectral bands with short wavelengths, differ across leaf layers and disease stages. The results of this study indicate that the main indicators for monitoring VW in cotton are those related to chlorophyll degradation, carotenoid cycling, moisture content, fluorescence, and anthocyanins. The relevant spectral bands primarily include the blue, green, red-edge, and parts of the near-infrared region, particularly the absorption valleys affected by moisture content. These findings align with previous studies on crop diseases, which have established that visible and near-infrared bands are sensitive bands for identifying different crop diseases (Xie et al., 2018; Zhang et al., 2019; Lassalle, 2021; Feng et al., 2022; Shafik et al., 2023).

At five-levels cotton VW severity monitoring, important signatures for the top leaf layer include Ant_Gitelson_, PRI_570_, PSDNb, and BF, while the middle leaf layer is characterized by B, PRI_600_, and Ant_Gitelson_, all of which are closely associated with vegetation pigments. Plant physiology research indicates that chlorophyll degradation and anthocyanin increase occur in leaves infected by VW, mainly as a protective response to damage caused by the pathogen (Camino et al., 2021). In the top and middle leaf layers, exposure to higher light intensity and increased photosynthetic activity leads to more pronounced changes in these physiological parameters, making chlorophyll degradation and anthocyanin-related indices more effective for VW monitoring. Blue band-related indices (e.g., BF, B, LIC) and PRI derivatives (e.g., PRI_570_, PRI_600_) play a significant role in monitoring cotton VW, consistent with findings from previous researches (Calderón et al., 2015). These features are important across the top, middle, and bottom leaf layers. The infection of VW typically leads to chlorophyll degradation, with significant carotenoid changes, and a reduction in photosynthetic efficiency, which are driven by pathogen-host interactions but not influenced by leaf position. As a result, indices related to chlorophyll and carotenoid absorption in the blue band, along with photosynthetic efficiency-related indices, remain important across different leaf layers.

In early disease monitoring, key features identified differ somewhat from those at five disease severity levels. Chlorophyll fluorescence emission related indices, such as PRI_1_ and CUR, play a significant role during the sightly infected stage, particularly in the top and middle layers. Fluorescence, as a by-product of photosynthesis, is closely tied to photosynthetic efficiency and photoprotection mechanisms, with potential to rapidly reflect plant stress states. In cotton plants, the average net photosynthetic rate decreases progressively from the upper to the lower leaves within the same growth stage (Li, 2007). In the slightly infected stage, upper leaves experience reversible inactivation of PSII reaction centers. Their exposure to higher light intensity further stimulates photoprotection mechanisms, including carotenoid cycling and non-photochemical quenching (NPQ) (Ruban, 2016), which leads to significant changes in fluorescence emissions. Similarly, middle leaves, benefiting from the cotton plant’s spiral leaf arrangement and canopy structure designed to optimize light utilization, also receive substantial light. This sufficient light exposure, during the slightly infected stages of disease, activates photoprotection mechanisms and results in significant changes in fluorescence emissions. In contrast, bottom leaves exposed to less light, exhibit weaker photoprotection changes, the fluorescence signals in these leaves are weaker and less responsive to early disease stress, making fluorescence-related indices less effective for monitoring early-stage disease in the lower canopy. However, the lower canopy exhibits a distinct advantage in early VW monitoring through water content-related indices (e.g., WSCT). Upper leaves, with stronger physiological activity, dynamically regulate water loss by closing stomata and reducing transpiration under early VW stress, thereby retaining water reserves (Pascual et al., 2010). In contrast, bottom leaves lack such regulatory capacity, leading to more pronounced water content variations during early infection.

It is noteworthy that certain bands play a critical role in monitoring cotton VW across different leaf positions and stages of disease development, particularly the blue and red-edge bands. In this study, a substantial number of features containing information from the blue and red-edge bands were identified as important for monitoring cotton VW. The red-edge band reflects both biochemical and biophysical factors, with its position shifting as chlorophyll declines and near-infrared reflectance rises due to structural changes (Barnes et al., 1992; PenUelas et al., 1995). It is consistently recognized as an effective wavelength for cotton VW monitoring (Yang et al., 2022b). However, this study explicitly indicates that, compared to the red-edge band, indicators related to the blue band, such as BF, B, BRI, and LIC, consistently prove to be highly significant for monitoring wilt severity across all stages of disease development, regardless of leaf layers. Also, these indicators demonstrate particularly strong performance in early monitoring. The blue band is highly sensitive to early disease stress due to changes in chlorophyll and carotenoid absorption. In the 450–499 nm region, chlorophyll strongly absorbs light, but its degradation during infection reveals carotenoid absorption features (Hennessy et al., 2020). This dual sensitivity to chlorophyll reduction and carotenoid prominence makes the blue band highly effective for monitoring subtle physiological changes during the slightly infected stage of disease. The blue band has consistently been considered as highly affected by disease stress at both the leaf and canopy scales (Poblete et al., 2020). Research on early monitoring of VW in olive trees has identified the blue/green index (BGI_1_) and the blue/red indices (LIC_3_ and BRI_1_) as robust indicators for wilt monitoring (Calderón et al., 2015). The results of this study also confirm previous findings, specifically that the blue/red spectral index (i.e., BRI) exhibits differences between asymptomatic and early-stage infected vegetation (Zarco-Tejada et al., 2018; Poblete et al., 2020; Camino et al., 2021; Watt et al., 2023). This conclusion aligns with multiple studies on olive tree wilt caused by *Verticillium*, which share similar pathogenic bacteria, but has been overlooked in existing research on cotton VW monitoring. This study achieved this discovery by detecting subtle changes in cotton leaf characteristics during the early stages of symptom onset.

In addition to constructing vegetation indices, this study extends beyond vegetation index construction by systematically analyzing morphological characteristics across the full spectral domain. Thirteen signal features were extracted for monitoring cotton VW from a novel perspective. The results indicate that the signal features play a significant role in cotton VW monitoring, especially in middle and bottom layer. Specifically, entropy and skewness are crucial for disease monitoring, ranking fourth and second in importance for the middle and bottom layers, respectively, during the slightly infected stage. Entropy is crucial for the middle layer, as it reflects the more complex reflectance changes resulting from the combined effects of phototropism and disease-induced structural and biochemical alterations. For the bottom leaves, spectral differences in the visible and near-infrared regions are greater than in the shortwave infrared for leaves at different disease severity levels, which results in differences in the asymmetry of spectral distributions across disease stages, making skewness an important feature. These signal features demonstrate potential for early-stage monitoring of cotton VW.

### 4.3 Limitations and future prospects

Many studies have explored the combination of thermal infrared, RGB, leaf imaging spectra, and PSR point spectra for multi-source remote sensing data to collaboratively diagnose diseases, which is a popular approach in the field of disease monitoring (Zarco-Tejada et al., 2018; Camino et al., 2021). Some research on cotton VW has attempted to monitor the disease from the image and spectrum perspectives, respectively (Kang et al., 2022). However, the effectiveness of integrating spectral data and imagery for collaborative monitoring of cotton VW remains unclear. Existing studies have demonstrated the advantages of spatial-spectral combination models for plant trait estimation, suggesting that fusing images and spectral data can enhance robustness and accuracy (Li et al., 2020; Räsänen et al., 2020; Xu et al., 2020). Spatial information of images was not considered in this study, and further research should investigate whether combining image and spectral features could improve the early-stage monitoring accuracy of cotton VW.

This study demonstrates the capability of timely and accurate diagnosis of leaf symptoms using *in situ* ground-based hyperspectral data under natural solar illumination. The method, which captures whole-leaf reflectance spectra, is fast, simple, and representative in practice. Moreover, it offers potential for adaptation across various sensing platforms, providing technical support for field applications. However, environmental and plant growth conditions in the field, which can interfere with remote sensing monitoring of diseases, have not yet been considered. Further research should focus on time-series spectral data to assess the plant’s growing status in relation to environmental effects and disease infection. This will provide a theoretical basis and methods for crop disease monitoring in precision agriculture.

## 5 Conclusion

This study focuses on investigating the impact of the vertical stratification of cotton leaves on the early monitoring of VW. *In-situ* hyperspectral data of cotton leaves were utilized to evaluate the performance of various feature selection algorithms and machine learning models for monitoring VW. It further analyzed the impact of leaf position and disease severity on monitoring accuracy, identifying key features for VW monitoring, particularly at early stages, and determining sensitive spectral bands. By comparing various feature selection methods and machine learning model combinations, RF feature selection combined with LightGBM demonstrated the best monitoring performance. Applying this optimal algorithm to different leaf layers for VW severity monitoring, the bottom layer leaves achieved the highest accuracy, with an accuracy of 0.91. Early-stage monitoring (SL=0-2, including healthy, slightly infected, and mildly infected stages) demonstrated higher accuracy in the bottom layer leaves compared to the middle and top layers, emphasizing the bottom leaves’ superior capability for early monitoring. In contrast, late-stage monitoring (SL=3-4, including moderately- and severely-infected stages) achieved high accuracy across all layers. In this study, blue and red-edge spectral bands were identified as critical for VW monitoring, with blue bands playing a particularly important role in early-stage monitoring-an aspect often overlooked in previous studies. This study provides technical support for timely, non-destructive, rapid, and accurate field-based monitoring of cotton VW. Early monitoring of infection in bottom layer leaves facilitates prompt intervention during the initial stages of pathogen invasion. Using spectral reflectance, this study discusses the early detection capability of cotton Verticillium wilt from the perspective of leaf stratification, considering the bottom-up disease progression mechanism. It focuses on analyzing the monitoring characteristics of the bottom leaves during the early stages of disease onset in cotton plants, thereby promoting the application of portable hyperspectral devices for precise early monitoring of cotton Verticillium wilt. Future work will focus on analyzing the temporal dynamics of leaf spectral changes, particularly in the blue and red-edge bands, as the disease progresses. Additionally, investigating the influence of different leaf positions on canopy-level spectral monitoring will be essential to determine the timeliness of monitoring VW infections using canopy-based remote sensing.

## Data Availability

The original contributions presented in the study are included in the article/supplementary material. Further inquiries can be directed to the corresponding author.
